# Supplementary material for: HeLa Cervical Cancer Cells Are Maintained by Nephronophthisis 3-Associated Primary Cilium Formation via ROS-Induced ERK and HIF-1α Activation under Serum-Deprived Normoxic Condition
Source: Int J Mol Sci. 2022 Nov 22;23(23):14500. doi: 10.3390/ijms232314500 (PMC9739938; doi:10.3390/ijms232314500)
Supplement: Supplementary file 1 [file ijms-23-14500-s001.zip › ijms-1978192-supplementary.pdf]

HeLa cervical cancer cells are maintained by nephronophthisis 3-associated primary cilium formation via ROS-induced ERK and HIF-1α activation under serum-deprived normoxic condition

Jae-Wook Lee, Jun Yeong Cho, Pham Xuan Thuy and Eun-Yi Moon *

Department of Integrative Bioscience and Biotechnology, Sejong University, Seoul 05006, Republic of Korea

* Corresponding author: eunyimoon@sejong.ac.kr

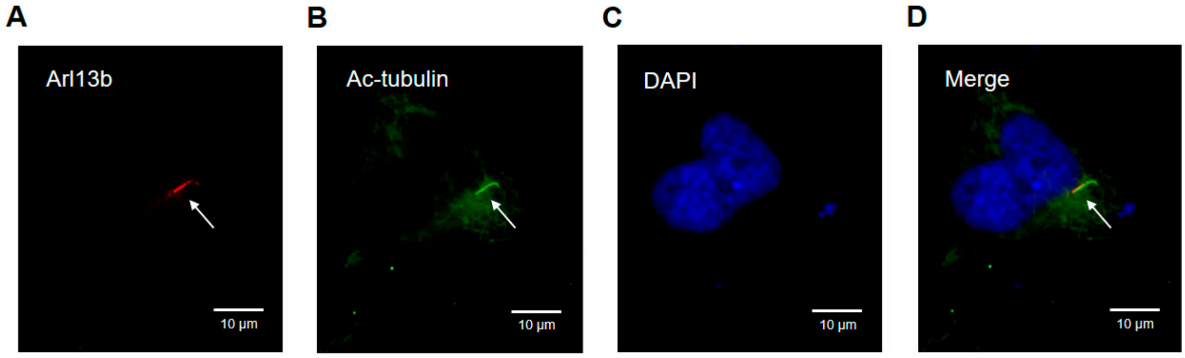

Supplementary Figure S1. Cilium was detected by immunostaining. HeLa cells were grown on coverslip. Cells were fixed with 4% paraformaldehyde for 10 min, washed three times with cold PBS, and permeabilized with 0.1% (v/v) Triton X-100 for 10 min. After washing three times, cells were incubated with anti-Arl13b antibodies (A) and anti-acetylated (Ac) tubulin antibodies (B) for 2 h at room temperature. After washing three times, cells were incubated with goat anti-rabbit IgG-Alexa 568 (A) and/or chicken anti-mouse IgG-Alexa 488 (B) for 1h at room temperature. Nucleus was visualized by staining cells with DAPI (C). After washing with PBS, cells were mounted on glass slide. Primary cilia were observed and photographed at 1,000 x magnification under a fluorescence microscope. Arl13b and Ac-tubulin respectively detected by Alexa 568 (red) and Alexa 488 (green) were merged in cilium (D). White arrows indicated primary cilia.

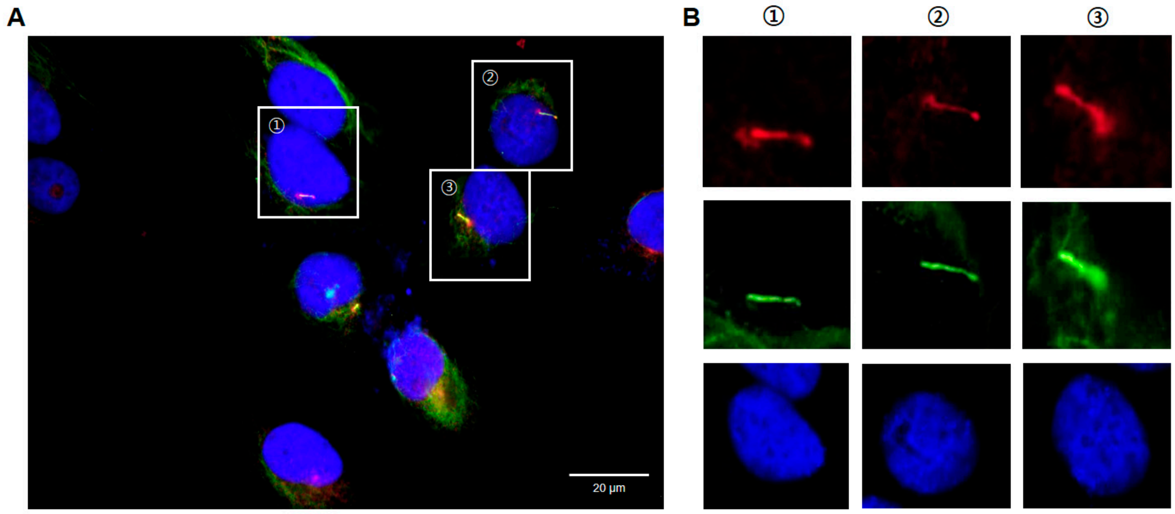

Supplementary Figure S2. Cilium was detected by immunostaining. HeLa cells were grown on coverslip and treated with 4 mM sodium butyrate for 48 h. Cells were fixed with 4% paraformaldehyde for 10 min, washed three times with cold PBS, and permeabilized with 0.1% (v/v) Triton X-100 for 10 min. After washing three times, cells were incubated with anti-Arl13b antibodies (red) and anti-acetylated tubulin antibodies (green) for 2 h at room temperature. After washing three times, cells were incubated with goat anti-rabbit IgG-Alexa 568 (red) and/or chicken anti-mouse IgG-Alexa 488 (green) for 1h at room temperature. Nucleus was visualized by staining cells with DAPI (blue). After washing with PBS, cells were mounted on glass slide. Primary cilia were observed and photographed at 1,000 x magnification under a fluorescence microscope. Arl13b and Ac-tubulin respectively detected by Alexa 568 and Alexa 488 were merged in cilium (A). Each primary cilium in white boxes was shown by the separate images of each channel (B).
